# Supplementary material for: Screening of Anisakis-Related Allergies and Associated Factors in a Mediterranean Community Characterized by High Seafood Consumption
Source: Foods. 2024 Sep 5;13(17):2821. doi: 10.3390/foods13172821 (PMC11395279; doi:10.3390/foods13172821)
Supplement: Supplementary file 1 [file foods-13-02821-s001.zip › foods-3159908-supplementary.pdf]

Supplementary Table S1. Answers to questionnaire questions

Questions	N (%)
**Contact with raw/marinated fish?**	
**No**	8 (3.09%)
**Yes**	251 (96.9%)
**If yes to question 1, intake as food?**	
**No**	12 (4.63%)
**Yes**	247 (95.4%)
**Food: estimated frequency of consumption last year?**	
**at least once a year**	44 (17.7%)
**at least once a month**	204 (82.3%)
**If yes to question 1, skin contact?**	
**No**	18 (7.11%)
**Yes**	235 (92.9%)
**Skin contact: estimated frequency last year?**	
**at least once a year**	33 (13.7%)
**at least once a month**	208 (86.3%)
**Do you use gloves when handling fish at home?**	
**Never**	233 (90.0%)
**Occasionally**	17 (6.56%)
**Always**	9 (3.47%)
**Have you ever had occupations that brought you into direct contact with fish or fish derivatives?**	
**No**	238 (91.9%)
**Yes**	21 (8.11%)
**Which is your current work activity?**	
**Housewife or unemployed person**	95 (36.7%)
**more**	46 (17.8%)
**Clerk or Worker**	44 (17.0%)
**Catering**	13 (5.02%)
**Students or Health Care**	61 (23.6%)
**Have you ever had allergic symptoms?**	
**No**	3 (1.16%)
**Yes**	256 (98.8%)
**asthma (1):**	
**No**	236 (91.1%)
**Yes**	23 (8.88%)
**rhinitis (2):**	
**No**	187 (72.2%)
**Yes**	72 (27.8%)
**conjunctivitis (3):**	
**No**	251 (96.9%)
**Yes**	8 (3.09%)
**brief urticaria (4):**	
**No**	143 (55.2%)
**Yes**	116 (44.8%)
**urticaria > 6 months (5):**	
**No**	227 (87.6%)
**Yes**	32 (12.4%)

**angioedema (6):**	
**No**	203 (78.4%)
**Yes**	56 (21.6%)
**other cutaneous (7):**	
**No**	227 (87.6%)
**Yes**	32 (12.4%)
**anaphylaxis (8):**	
**No**	254 (98.1%)
**Yes**	5 (1.93%)
**Which is the frequency of symptoms in the last year?**	
**<10 times**	126 (49.0%)
**10-30 times**	74 (28.8%)
**> 30 times**	57 (22.2%)
**If yes, how long have you had the allergy (years)?**	
**Mean (SD)**	10.2 (10.7)
**Have you ever been diagnosed with chronic gastrointestinal diseases (Crohn's disease, ulcerative rectocolitis, indeterminate colitis)?**	
**No**	257 (99.2%)
**Yes**	2 (0.77%)
**Any presence of one the following symptoms?**	
**No**	28 (10.8%)
**Yes**	231 (89.2%)
**Diarrhoea:**	
**No**	163 (62.9%)
**Yes**	96 (37.1%)
**Abdominal pain:**	
**No**	128 (49.4%)
**Yes**	131 (50.6%)
**Melena:**	
**No**	241 (93.1%)
**Yes**	18 (6.95%)
**Nausea:**	
**No**	144 (55.6%)
**Yes**	115 (44.4%)
**Constipation:**	
**No**	119 (45.9%)
**Yes**	140 (54.1%)
**Fever:**	
**No**	193 (74.5%)
**Yes**	66 (25.5%)
**Ematemesis:**	
**No**	255 (98.5%)
**Yes**	4 (1.54%)
**Vomiting:**	
**No**	203 (78.4%)
**Yes**	56 (21.6%)
**Which is the frequency of symptoms in the last year?**	
**<10 times**	133 (57.8%)

**10-30 times**	77 (33.5%)
**> 30 times**	20 (8.70%)
**Have you ever carried out a parasitological stool test?**	
**No**	178 (68.7%)
**Yes**	81 (31.3%)
**How often do you eat red or processed meat (cooked ham, ham, bresaola, salami, mortadella, bacon, sausages, cotechino, frankfurters)?**	
**less than twice a month**	45 (17.4%)
**at least twice a month**	214 (82.6%)
**How often do you eat smoked food (bacon, Prague ham, bacon, or other smoked meat)?**	
**less than twice a month**	161 (62.2%)
**at least twice a month**	98 (37.8%)
**How often do you drink alcohol (beer, wine)?**	
**less than twice a month**	188 (72.6%)
**at least twice a month**	71 (27.4%)
**How often do you drink spirits?**	
**less than twice a month**	219 (84.6%)
**at least twice a month**	40 (15.4%)
**Do you currently smoke (cigarettes or tobacco, cigars, electronic cigarettes)?**	
**No**	192 (74.1%)
**SI**	67 (25.9%)
**If No, have you smoked in the past?**	
**I have smoked IN THE PAST, now I have stopped**	62 (38.0%)
**I have never smoked**	101 (62.0%)
**If YES, at what age did you start smoking?**	
**Mean (SD)**	16.8 (3.93)
**If YES, at what age did you stop?**	
**Mean (SD)**	31.3 (13.3)
	**SD = Standard Deviatio**

Supplementary Table S2. Univariate comparison of study variables and positivity to specific Anisakis IgE and BAT.

	Total (%)	Anisakis kUA/l<0.1 n. (%)	Anisakis kUA/l≥0.1 n. (%)	OR [IC95%]	p-value	BAT <15% n. (%)	BAT ≥15% n. (%)	OR [IC95%]	p-value
	**N=259 (100)**	**N=211**	**N=48**			**N=247**	**N=12**		
**Age media (SD)**	40.8 (20.5)	39.4 (19.6)	46.6 (23.2)	1.02 [1.01;1.03]	**0.030**	40.1 (20.4)	54.3 (19.2)	1.04 [1.00;1.07]	**0.024**
**Sex**									
**Male**	88 (34.0)	61 (28.9)	27 (56.2)	Ref.	Ref.	81 (32.8)	7 (58.3)	Ref.	Ref.
**Female**	171 (66.0)	150 (71.1)	21 (43.8)	0.32 [0.17;0.61]	**<0.001**	166 (67.2)	5 (41.7)	0.35 [0.10;1.17]	0.087
**Anisakis (kUA/l ≥0.1)**									
**Negative**	211 (81.5)				-	211 (85.4)	0 (0.0)	Ref.	Ref.
**Positive**	48 (18.5)				-	36 (14.6)	12 (100.0)	∞	**<0.001**
**Ascaris (kUA/l ≥0.1)**									
**Negative**	222 (85.7)	211 (100.0)	11 (22.9)	Ref.	Ref.	220 (89.1)	2 (16.7)	Ref.	Ref.
**Positive**	37 (14.3)	0 (0.0)	37 (77.1)	∞	**<0.001**	27 (10.9)	10 (83.3)	37.5 [9.1;277.0]	**<0.001**
**Tropomyosin (kUA/l ≥0.1)**									
**Negative**	249 (96.1)	211 (100.0)	38 (79.2)	Ref.	Ref.	241 (97.6)	8 (66.7)	Ref.	Ref.
**Positive**	10 (3.9)	0 (0.0)	10 (20.8)	∞	**<0.001**	6 (2.4)	4 (33.3)	19.5 [4.11;86.2]	**<0.001**
**Education level:**									
**Primary**	43 (16.6)	31 (14.3)	12 (25.0)	Ref.	Ref.	40 (16.2)	3 (25.0)	Ref.	Ref.
**Lower secondary education.**	94 (36.3)	77 (36.5)	17 (35.4)	0.57 [0.24;1.37]	0.205	90 (36.4)	4 (33.3)	0.59 [0.12;3.32]	0.522
**Upper secondary education**	95 (36.7)	81 (38.4)	14 (29.2)	0.45 [0.18;1.10]	0.079	92 (37.2)	3 (25.0)	0.44 [0.07;2.64]	0.350
**Degree**	27 (10.4)	22 (10.4)	5 (10.4)	0.60 [0.17;1.90]	0.394	25 (10.1)	2 (16.7)	1.09 [0.12;7.63]	0.932
**Area of Residence:**									
**Inland area**	47 (18.1)	38 (18.0)	9 (18.8)	Ref.	Ref.	45 (18.2)	2 (16.7)	Ref.	Ref.
**Marine area**	80 (30.9)	69 (32.7)	11 (22.9)	0.67 [0.25;1.83]	0.432	76 (30.8)	4 (33.3)	1.14 [0.20;9.55]	0.884
**Urban area (Palermo - Catania - Messina)**	132 (51.0)	104 (49.3)	28 (58.3)	1.13 [0.50;2.75]	0. 783	126 (51.0)	6 (50.0)	1.02 [0.22;7.95]	0.979

	Total (%)	Anisakis kUA/l<0.1 n. (%)	Anisakis kUA/l≥0.1 n. (%)	OR [IC95%]	p-value	BAT <15% n. (%)	BAT ≥15% n. (%)	OR [IC95%]	p-value
**How many years have you had your current residence?**									
**Less than 10 years**	26 (10.0)	23(10.9)	3 (6.2)	Ref.	Ref.	24 (9.7)	2 (16.7)	Ref.	Ref.
**More than 10 years**	233 (90.0)	188 (89.1)	45 (93.8)	1.76 [0.57;7.97]	0.352	223 (90.3)	10 (83.3)	0.51 [0.12;3.81]	0.451
**Question 1: Contact with raw/marinated fish?**									
**No**	8 (3.1)	6(2.8)	2(4.2)	Ref.	Ref.	7 (2.8)	1 (8.3)	Ref.	Ref.
**Yes**	251 (96.9)	205 (97.2)	46 (95.8)	0.64 [0.14;4.99]	0.623	240 (97.2)	11 (91.7)	0.29 [0.04;7.92]	0.367
**If yes to question 1 , intake as food?**									
**No**	12 (4.6)	5(2.4)	7(14.6)	Ref.	Ref.	9 (3.6)	3 (25.0)	Ref.	Ref.
**Yes**	247 (95.4)	206 (97.6)	41 (85.4)	0.14 [0.04;0.49]	**0.002**	238 (96.4)	9 (75.0)	0.11 [0.03;0.61]	**0.014**
**Food: estimated frequency of consumption last year: (3 missing values)**									
**at least once a year**	44 (17.7)	35(17.0)	9 (21.4)	Ref.	Ref.	40 (16.7)	4 (44.4)	Ref.	Ref.
**at least once a month**	204 (82.3)	171 (83.0)	33 (78.6)	0.74 [0.34;1.79]	0.493	199 (83.3)	5 (55.6)	0.25 [0.05;1.33]	0.055
**If yes to question 1, skin contact?**									
**No**	18 (7.11)	10 (4.9)	8 (16.7)	Ref.	Ref.	15 (6.2)	3 (25.0)	Ref.	Ref.
**Yes**	235 (92.9)	195 (95.1)	40 (83.3)	0.16 [0.09;0.72]	**0.011**	226 (93.8)	9 (75.0)	0.20 [0.05;0.99]	**0.044**
**Skin contact: estimated frequency last year:**									
**at least once a year**	33 (13.7)	28 (14.1)	5 (11.6)	Ref.	Ref.	31 (13.4)	2 (20.0)	Ref.	Ref.
**at least once a year**	208 (86.3)	170 (85.9)	38 (88.4)	1.22 [0.47;3.86]	0.696	200 (86.6)	8 (80.0)	0.62 [0.12-6.27]	0.631
**Do you use gloves when handling fish at home?**									
**Never**	233 (90.0)	191 (90.5)	42 (87.5)	Ref.	Ref.	223 (90.3)	10 (83.4)	Ref.	Ref.
**Occasionally**	17 (6.56)	13 (6.2)	4 (8.3)	1.43 [0.38;4.34]	0.566	16 (6.4)	1 (8.3)	1.56 [0.06;9.12]	0.713
**Always**	9 (3.47)	7 (3.3)	2 (4.2)	1.36 [0.18;6.07]	0.722	8 (3.2)	1 (8.3)	3.07 [0.11;20.0]	0.404
**Have you ever had occupations that brought you into direct contact with fish or fish derivatives?**									

	Total (%)	Anisakis kUA/l<0.1 n. (%)	Anisakis kUA/l≥0.1 n. (%)	OR [IC95%]	p-value	BAT <15% n. (%)	BAT ≥15% n. (%)	OR [IC95%]	p-value
**No**	238 (91.9)	195 (92.4)	43 (89.6)	Ref.	Ref.	227 (91.9)	11 (91.7)	Ref.	Ref.
**Yes**	21 (8.1)	16 (7.6)	5 (10.4)	1.44 [0.44;3.96]	0.515	20 (8.1)	1 (8.3)	1.16 [0.05;6.54]	0.898
**Describe your current work activity:**									
**Housewife or unemployed person**	95 (36.7)	78 (37.0)	17 (35.4)	Ref.	Ref.	89 (36.0)	6 (50.0)	Ref.	Ref.
**other**	46 (17.8)	39 (18.5)	7 (14.6)	0.83 [0.30;2.13]	0.711	45 (18.2)	1 (8.3)	0.37 [0.01;2.34]	0.329
**Clerk or Worker**	44 (17.0)	32 (15.2)	12 (25.0)	1.72 [0.72;4.02]	0.219	42 (17.0)	2 (16.7)	0.74 [0.09;3.50]	0.724
**Catering**	13 (5.0)	10 (4.7)	3 (6.2)	1.41 [0.28;5.34]	0.644	11 (4.45)	2 (16.7)	2.77 [0.33;14.4]	0.301
**Students or Health Care**	61 (23.6)	52 (24.6)	9 (18.8)	0.80 [0.32;1.91]	0.622	60 (24.3)	1 (8.3)	0.28 [0.01;1.74]	0.193
**Have you ever had allergic symptoms?**									
**No**	3 (1.2)	2 (0.9)	1 (2.1)	Ref.	Ref.	3 (1.2)	0 (0.0)	Ref.	Ref.
**Yes**	256 (98.8)	209 (99.1)	47 (97.9)	0.42 [0.03;13.50]	0.550	244 (98.8)	12 (100)	+∞ [0.02;+ ∞]	1.000
**asthma:**									
**No**	236 (91.1)	194 (91.9)	42 (87.5)	Ref.	Ref.	225 (91.1)	11 (91.7)	Ref.	Ref.
**Yes**	23 (8.9)	21 (8.1)	6 (12.5)	1.65 [0.56;4.28]	0.343	22 (8.9)	1 (8.33)	1.04 [0.04;5.85]	0.970
**rhinitis:**									
**No**	187 (72.2)	150 (71.1)	37 (77.1)	Ref.	Ref.	177 (71.7)	10 (83.3)	Ref.	Ref.
**Yes**	72 (27.8)	61 (28.9)	11 (22.9)	0.74 [0.34;1.51]	0.413	70 (28.3)	2 (16.7)	0.54 [0.07;2.14]	0.411
**conjunctivitis:**									
**No**	251 (96.9)	206 (97.6)	45 (93.8)	Ref.	Ref.	239 (96.8)	12 (100)	Ref.	Ref.
**Yes**	8 (3.1)	5 (2.4)	3 (6.2)	2.78 [0.52;12.3]	0.210	8 (3.24)	0 (0.0)	0.00 [0.0;13.19]	1.000
**brief urticaria:**									
**No**	143 (55.2)	117 (55.5)	26 (54.2)	Ref.	Ref.	137 (55.5)	6 (50.0)	Ref.	Ref.
**Yes**	116 (44.8)	94 (44.5)	8 (45.8)	1.05 [0.56;1.98]	0.871	110 (44.5)	6 (50.0)	1.24 [0.37;4.19]	0.718
**urticaria > 6 months:**									
**No**	227 (87.6)	186 (88.2)	41 (85.4)	Ref.	Ref.	215 (87.0)	12 (100)	Ref.	Ref.
**Yes**	32 (12.4)	25 (11.8)	7 (14.6)	1.29 [0.48;3.06]	0.596	32 (13.0)	0 (0.0)	0.00 [0.00;2.56]	0.372
**angioedema:**									

	Total (%)	Anisakis kUA/l<0.1 n. (%)	Anisakis kUA/l≥0.1 n. (%)	OR [IC95%]	p-value	BAT <15% n. (%)	BAT ≥15% n. (%)	OR [IC95%]	p-value
**No**	203 (78.4)	165 (78.2)	38 (79.2)	Ref.	Ref.	192 (77.7)	11 (91.7)	Ref.	Ref.
**Yes**	56 (21.6)	46(21.8)	10 (20.8)	0.95 [0.42;2.00]	0.903	55 (22.3)	1 (8.3)	0.36 [0.01;1.93]	0.275
**other cutaneous:**									
**No**	227 (87.6)	187 (88.6)	40 (83.3)	Ref.	Ref.	218 (88.3)	9 (75.0)	Ref.	Ref.
**Yes**	32 (12.4)	24 (11.4)	8 (16.7)	1.57 [0.62;3.65]	0.327	29 (11.7)	3 (25.0)	2.57 [0.52;9.41]	0.220
**anaphylaxis:**									
**No**	254 (98.1)	206 (97.6)	48 (100)	Ref.	Ref.	242 (98.0)	12 (100)	Ref.	Ref.
**Yes**	5 (1.9)	5 (2.4)	0 (0.0)	0.00 [0.00;4.8]	0.588	5 (2.02)	0 (0.0)	0.00 [0.0;24.34]	1.000
**Frequency of symptoms/last year**									
**<10 times**	126 (49.0)	102 (48.6)	24 (51.1)	Ref.	Ref.	119 (48.6)	7 (58.3)	Ref.	Ref.
**> 30 times**	57 (22.2)	49 (23.3)	8 (17.0)	0.70 [0.28;1.63]	0.422	54 (22.0)	3 (25.0)	0.97 [0.19;3.74]	0.967
**10-30 times**	74 (28.8)	59 (28.1)	15 (31.9)	1.08 [0.52;2.22]	0.829	72 (29.4)	2 (16.7)	0.50 [0.07;2.19]	0.380
**If yes, how long have you had the allergy (years)?**									
**Mean (SD)**	10.2 (10.7)	9.80 (10.1)	12.0 (13.0)	1.02 [0.99;1.05]	0.218	9.89 (10.3)	16.2 (16.7)	1.04 [1.00;1.09]	0.055
**Have you ever been diagnosed with chronic gastrointestinal diseases (Crohn's disease, ulcerative rectocolitis, indeterminate colitis)?**									
**No**	257 (99.2)	209 (99.1)	48 (100)	Ref.	Ref.	245 (99.2)	12 (100)	Ref.	Ref.
**Yes**	2 (0.8)	2 (0.9)	0 (0.0)	0.00 [0.00;23.55]	1.000	2 (0.81)	0 (0.0)	0.00 [0.0;113.5]	1.000
**Presence of the following symptoms: diarrhoea, abdominal pain, melena, nausea, constipation, fever, haematemesis or vomiting**									
**No**	28 (10.8)	24 (11.4)	4 (8.3)	Ref.	Ref.	28 (11.3)	0 (0.0)	Ref.	Ref.
**Yes**	231 (89.2)	187 (88.6)	44 (91.7)	1.37 [0.49;4.97]	0.572	219 (88.7)	12 (100)	+∞ [0.33;+∞]	0.373
**Diarrhoea:**									
**No**	163 (62.9)	130 (61.6)	33 (68.8)	Ref.	Ref.	156 (63.2)	7 (58.3)	Ref.	Ref.
**Yes**	96 (37.1)	81 (38.4)	15 (31.2)	0.73 [0.37;1.42]	0.363	91 (36.8)	5 (41.7)	1.23 [0.35;4.07]	0.733

	Total (%)	Anisakis kUA/l<0.1 n. (%)	Anisakis kUA/l≥0.1 n. (%)	OR [IC95%]	p-value	BAT <15% n. (%)	BAT ≥15% n. (%)	OR [IC95%]	p-value
**Abdominal pain:**									
**No**	128 (49.4)	104 (49.3)	24 (50.0)	Ref.	Ref.	123 (49.8)	5 (41.7)	Ref.	Ref.
**Yes**	131 (50.6)	107 (50.7)	24(50.0)	0.97 [0.52;1.83]	0.930	124 (50.2)	7 (58.3)	1.38 [0.42;4.90]	0.600
**Melena:**									
**No**	241 (93.1)	198 (93.8)	43 (89.6)	Ref.	Ref.	229 (92.7)	12 (100)	Ref.	Ref.
**Yes**	18 (6.9)	13 (6.16)	5 (10.4)	1.80 [0.54;5.11]	0.316	18 (7.29)	0 (0.0)	0.00 [0.00;5.03]	1.000
**Nausea:**									
**No**	144 (55.6)	114 (54.0)	30 (62.5)	Ref.	Ref.	138 (55.9)	6 (50.0)	Ref.	Ref.
**Yes**	115 (44.4)	97 (46.0)	18 (37.5)	0.71 [0.36;1.34]	0.292	109 (44.1)	6 (50.0)	1.26 [0.38;4.25]	0.697
**Constipation:**									
**No**	119 (45.9)	99 (46.9)	20 (41.7)	Ref.	Ref.	115 (46.6)	4 (33.3)	Ref.	Ref.
**Yes**	140 (54.1)	112 (53.1)	28 (58.3)	1.23 [0.66;2.36]	0.517	132 (53.4)	8 (66.7)	1.71 [0.51;6.77]	0.392
**Fever:**									
**No**	193 (74.5)	154 (73.0)	39 (81.2)	Ref.	Ref.	184 (74.5)	9 (75.0)	Ref.	Ref.
**Yes**	66 (25.5)	57 (27.0)	9 (18.8)	0.63 [0.27;1.34]	0.240	63 (25.5)	3 (25.0)	1.01 [0.21;3.57]	0.994
**Ematemesis:**									
**No**	255 (98.5)	207 (98.1)	48 (100)	Ref.	Ref.	243 (98.4)	12 (100)	Ref.	Ref.
**Yes**	4 (1.54)	4 (1.9)	0 (0.0)	0.00 [0.00;17.41]	1.000	4 (1.62)	0 (0.00)	0.00 [0.0;33.48]	1.000
**Vomiting:**									
**No**	203 (78.4)	169 (80.1)	34 (70.8)	Ref.	Ref.	196 (79.4)	7 (58.3)	Ref.	Ref.
**Yes**	56 (21.6)	42 (19.9)	14 (29.2)	1.66 [0.80;3.34]	0.172	51 (20.6)	5 (41.7)	2.75 [0.76;9.20]	0.116
**Frequency of symptoms/last year**									
**<10 times**	133 (57.8)	102 (54.8)	31 (70.4)	Ref.	Ref.	124 (56.9)	9 (75.0)	Ref.	Ref.
**10-30 times**	77 (33.5)	69 (37.1)	8 (18.2)	0.39 [0.16;0.86]	**0.019**	76 (34.9)	1 (8.3)	0.20 [0.01;1.14]	**0.075**
**> 30 times**	20 (8.7)	15 (8.1)	5 (11.4)	1.12 [0.33;3.18]	0.848	18 (8.26)	2 (16.7)	1.60 [0.21;7.06]	0.594
**Have you ever carried out a parasitological stool test?**									
**No**	178 (68.7)	151 (71.6)	27 (56.2)	Ref.	Ref.	171 (69.2)	7 (58.3)	Ref.	Ref.
**Yes**	81 (31.3)	60 (28.4)	21 (43.8)	1.95 [1.02;3.73]	**0.045**	76 (30.8)	5 (41.7)	1.62 [0.45;5.35]	0.441

	Total (%)	Anisakis kUA/l<0.1 n. (%)	Anisakis kUA/l≥0.1 n. (%)	OR [IC95%]	p-value	BAT <15% n. (%)	BAT ≥15% n. (%)	OR [IC95%]	p-value
**How often do you eat red or processed meat (cooked ham, ham, bresaola, salami, mortadella, bacon, sausages, cotechino, frankfurters)?**									
**less than twice a month**	45 (17.4)	34 (16.1)	11 (22.9)	Ref.	Ref.	42 (17.0)	3 (25.0)	Ref.	Ref.
**at least twice a month**	214 (82.6)	177 (83.9)	37 (77.1)	0.64 [0.30;1.44]	0.273	205 (83.0)	9 (75.0)	0.60 [0.17;2.92]	0.483
**How often do you eat smoked food (bacon, Prague ham, bacon, or other smoked meat)?**									
**less than twice a month**	161 (62.2)	134 (63.5)	27 (56.2)	Ref.	Ref.	155 (62.8)	6 (50.0)	Ref.	Ref.
**at least twice a month**	98 (37.8)	77 (36.5)	21 (43.8)	1.35 [0.71;2.56]	0.355	92 (37.2)	6 (50.0)	1.68 [0.50;5.66]	0.392
**How often do you drink alcohol? (beer, wine):**									
**less than twice a month**	188 (72.6)	157 (74.4)	31 (64.6)	Ref.	Ref.	182 (73.7)	6 (50.0)	Ref.	Ref.
**at least twice a month**	71 (27.4)	54 (25.6)	17 (35.4)	1.60 [0.80;3.10]	0.179	65 (26.3)	6 (50.0)	2.79 [0.82;9.44]	0.097
**How often do you drink spirits?**									
**less than twice a month**	219 (84.6)	175 (82.9)	44 (91.7)	Ref.	Ref.	210 (85.0)	9 (75.0)	Ref.	Ref.
**at least twice a month**	40 (15.4)	36 (17.1)	4 (8.3)	0.46 [0.13;1.23]	0.128	37 (15.0)	3 (25.0)	1.95 [0.40;7.03]	0.373
**Do you currently smoke (cigarettes or tobacco, cigars, electronic cigarettes)?**									
**No**	192 (74.1)	157 (74.4)	35 (72.9)	Ref.	Ref.	184 (74.5)	8 (66.7)	Ref.	Ref.
**Yes**	67 (25.9)	54 (25.6)	13 (27.1)	1.09 [0.52;2.17]	0.821	63 (25.5)	4 (33.3)	1.48 [0.37;4.99]	0.548
**If No, have you smoked in the past?**									
**I have smoked IN THE PAST, now I have stopped**	62 (38.0)	50 (37.9)	12 (38.7)	Ref.	Ref.	59 (38.1)	3 (37.5)	Ref.	Ref.
**I have never smoked**	101 (62.0)	82 (62.1)	19 (61.3)	0.96 [0.43;2.21]	0.926	96 (61.9)	5 (62.5)	1.01 [0.23;5.37]	0.995

	Total (%)	Anisakis kUA/l<0.1 n. (%)	Anisakis kUA/l≥0.1 n. (%)	OR [IC95%]	p-value	BAT <15% n. (%)	BAT ≥15% n. (%)	OR [IC95%]	p-value
**If YES, at what age did you start smoking?**									
**Mean (SD)**	16.8 (3.93)	16.8 (4.25)	17.2 (2.04)	1.03 [0.87;1.22]	0.745	16.8 (3.99)	16.7 (3.21)	0.99 [0.74;1.33]	0.938
**If YES, at what age did you stop?**									
**Mean (SD)**	31.3 (13.3)	31.0 (13.70)	32.8 (11.9)	1.01 [0.96;1.06]	0.677	31.5 (13.3)	27.3 (17.2)	0.97 [0.88;1.08]	0.596

SD = Standard Deviation
